# Methods used to meta-analyse results from interrupted time series studies: A methodological systematic review protocol

**DOI:** 10.12688/f1000research.22226.3

**Published:** 2020-12-11

**Authors:** Elizabeth Korevaar, Amalia Karahalios, Andrew B. Forbes, Simon L. Turner, Steve McDonald, Monica Taljaard, Jeremy M. Grimshaw, Allen C. Cheng, Lisa Bero, Joanne E. McKenzie

**Affiliations:** 1 School of Public Health and Preventive Medicine, Monash University, Melbourne, Victoria, 3004, Australia; 2 Clinical Epidemiology Program, Ottawa Hospital Research Institute, Ottawa, Ontario, K1Y 4E9, Canada; 3 School of Epidemiology, Public Health and Preventive Medicine, University of Ottawa, Ottawa, Ontario, K1N 6N5, Canada; 4 Department of Medicine, University of Ottawa, Ottawa, Ontario, K1H 8M5, Canada; 5 Infection Prevention and Healthcare Epidemiology Unit, Alfred Health, Melbourne, Victoria, 3004, Australia; 6 Faculty of Medicine and Health and Charles Perkins Centre, University of Sydney, Sydney, New South Wales, 2006, Australia

**Keywords:** Interrupted time series, meta-analysis, systematic review

## Abstract

**Background: **Systematic reviews are used to inform healthcare decision making. In reviews that aim to examine the effects of organisational, policy change or public health interventions, or exposures, evidence from interrupted time series (ITS) studies may be included. A core component of many systematic reviews is meta-analysis, which is the statistical synthesis of results across studies. There is currently a lack of guidance informing the choice of meta-analysis methods for combining results from ITS studies, and there have been no studies examining the meta-analysis methods used in practice. This study therefore aims to describe current meta-analysis methods used in a cohort of reviews of ITS studies.

**Methods:** We will identify the 100 most recent reviews (published between 1 January 2000 and 11 October 2019) that include meta-analyses of ITS studies from a search of eight electronic databases covering several disciplines (public health, psychology, education, economics). Study selection will be undertaken independently by two authors. Data extraction will be undertaken by one author, and for a random sample of the reviews, two authors. From eligible reviews we will extract details at the review level including discipline, type of interruption and any tools used to assess the risk of bias / methodological quality of included ITS studies; at the meta-analytic level we will extract type of outcome, effect measure(s), meta-analytic methods, and any methods used to re-analyse the individual ITS studies. Descriptive statistics will be used to summarise the data.

**Conclusions: **This review will describe the methods used to meta-analyse results from ITS studies. Results from this review will inform future methods research examining how different meta-analysis methods perform, and ultimately, the development of guidance.

## Introduction

Systematic reviews aim to collate and synthesise all available evidence on a particular topic. They are used to inform healthcare decision making, either directly, or through their inclusion in knowledge tools such as clinical practice guidelines. Many reviews examining the effects of clinical interventions are appropriately limited in scope to inclusion of randomised trials. However, in reviews where the aim is to examine the effects of organisational, policy change or public health interventions or exposures (e.g. chemical exposures), evidence from non-randomised studies may offer the only evidence, or provide important additional evidence to that gained from randomised trials ^1^.

Interrupted time series (ITS) studies are a type of non-randomised design in which measurements on a group of individuals (e.g. a community) are taken repeatedly both before and after an ‘interruption’ ^2^. The interruption may be intended (e.g. a government-implemented policy), although will not necessarily be initiated or designed by the ITS investigators (e.g. by researchers within a university) ^3^, or may be unintended (e.g. an exposure such as a natural disaster). The key benefit of the ITS design is that the period before the interruption can be used to estimate the underlying time trend. If modelled correctly, this before-trend can be projected into the post-interruption period to provide a counterfactual for what would have occurred in the absence of the interruption ^4^. ITS studies with controls (e.g. an internal or external control series, control outcome) may provide more certainty in causally attributing any observed effects to the interruption ^5^. Several effect estimates can be obtained from an ITS study to characterise both short and long-terms effects of the interruption (e.g. level change and slope change).

Meta-analysis is the statistical synthesis of results across studies leading to combined effect estimates ^6^. Meta-analysis (and its extensions) is a core component of many systematic reviews. The benefits of meta-analysis have long been established, including the ability to more precisely estimate effects, examine and quantify inconsistency of the effects across studies, and identify factors that may potentially modify the size of the effects ^7– 10^. Two approaches for meta-analysing results from ITS studies include the two-stage or one-stage approach ^11^. In the two-stage approach, effect estimates from each series are first computed, and these are then combined across series using a meta-analysis method (e.g. DerSimonian and Laird ^12^). In the one-stage approach, a single model including all series is fitted to simultaneously obtain the combined effect estimates ^11^. The one-stage approach requires the raw time series data to be available for all series, but has the proposed advantage of being more efficient since the data across all the series are used in estimating the effects ^11^.

For two-stage meta-analysis, a notable challenge is that many primary ITS studies are analysed incorrectly. For example, ITS studies may be analysed as though the study was a before-after design ^13^, or analysed as an ITS design, but without taking account of the correlation between observations over time (known as autocorrelation) ^14– 16^. The former is likely to result in estimates of the effect of the interruption that are biased, while the latter is likely to result in estimates of standard errors that are too small ^17^. Both have important implications for a two-stage meta-analysis in terms of bias, the weights that studies receive, and in turn, the precision of the combined estimate. A further challenge is that the effect measures chosen and reported by the primary study authors (e.g. level change) may not match those of interest to the systematic reviewer (e.g. slope change).

In some studies, the raw time series data may be available through extraction of data from graphs or their availability in tables ^6, 8, 11, 18^. In this circumstance, it may be possible to overcome some of the above challenges through re-analysis of the ITS studies by appropriately accounting for the design and autocorrelation, or re-analysing the raw data to obtain the desired effect measure for the meta-analysis when it differs from that reported in the primary study. These computed effects may then be combined using two-stage meta-analysis. Alternatively, each study’s raw data may be analysed in a single model using a one-stage meta-analysis approach ^6, 8^.

To our knowledge, there have been no reviews examining the approaches and methods used to meta-analyse effect estimates from ITS studies. In this review we therefore aim to: 1) investigate whether reviewers re-analyse primary ITS studies included in reviews, and if so, what re-analysis methods are used; 2) what meta-analysis methods are used; 3) what effect measures are used, and how completely the estimated combined effects are reported; and 4) what tools and domains are used to assess the risks of bias or methodological quality of the included ITS studies. Here, we report the planned design of our review, including the criteria that we will use to identify eligible studies, as well as the information we will extract and describe.

## Methods

### Overview

This study aims to identify and describe reviews that include meta-analyses of ITS studies. The reviews will be identified by searching several electronic databases including MEDLINE (Ovid), EMBASE (Ovid), Campbell Systematic Reviews, EconLit (EBSCOhost), 3ie, PsycINFO (Ovid), ERIC (ProQuest) and the Cochrane Database of Systematic Reviews (CDSR). Study selection will be undertaken independently by two authors; data extraction will be undertaken by one author, and for a minimum of 20% of randomly selected reviews, two authors. We will extract details at the systematic review level, including: discipline (public health, psychology, education, economics), type of interruption, assessment of risk of bias and methodological quality; and at the meta-analytic level: type of outcome, effect measure(s), meta-analytic methods, and any methods used to re-analyse the individual ITS studies. These aspects will be analysed and described using summary statistics, tables and figures.

### Eligibility criteria

Studies which meet our eligibility criteria (described below) will be included. We will not restrict inclusion of reviews based on discipline or any of the PICO elements (i.e. participants/populations, interventions/interruptions, comparators, or outcomes).

***Inclusion criteria.*** Studies meeting the following criteria will be included:

1. the study is a review that includes at least two ITS studies which meet the review authors’ definition of an ITS design; and
2. the review includes at least one meta-analysis of ITS studies.

Our definition of a ‘review’ is very broad. It includes systematic reviews, reviews of selected studies (i.e. between-study meta-analysis), and studies that combine multiple ITS across sites within the same study (i.e. within-study meta-analysis). We have opted for broad inclusion since our primary interest is in the meta-analysis methods, which apply regardless of the particular study design. We will not restrict the meta-analysis by approach, that is, we will include both one-stage and two-stage meta-analyses. We will only include meta-analyses that provide pooled estimates of model parameters that quantify the effect of the interruption.

***Exclusion criteria.*** Studies will be excluded if they meet one or more of the following criteria. The study is:

1. written in a language other than English;
2. a methodological review that describes or evaluates methods to synthesise results from ITS studies;
3. a review of ITS studies reported in a conference abstract, letter, book, or dissertation;
4. a protocol for a review of ITS studies; or
5. a stepped-wedge randomised trial.

Criterion 1 is included because we are not able to translate studies written in a language other than English due to resource constraints. Criterion 2 excludes methodological reviews that describe or evaluate methods to synthesise data from ITS studies, as our aim is to describe current statistical methods applied in practice.

### Search methods

Several databases will be searched to capture the broad range of disciplines that use the ITS study design. To capture reviews in health, we will search MEDLINE (Ovid), EMBASE (Ovid), Campbell Systematic Reviews, the CDSR and 3ie. For CDSR, we will directly search the ‘Characteristics of included studies’ table included in each systematic review for ITS studies. This will allow more specific identification of eligible reviews. The search of MEDLINE (Ovid) will also capture systematic reviews from the Joanna Briggs Institute Database of Systematic Reviews and Implementation Reports. To capture reviews in economics, we will search EconLit (EBSCOhost), and for psychology and education disciplines, we will search PsycINFO (Ovid) and ERIC (ProQuest).

Our search strategy has been informed by previous publications that have reviewed ITS studies ^14, 15, 19^. Reviews of ITS studies will be identified using terms adapted from the search strategies of these publications and then combined with terms to identify meta-analyses and systematic reviews. As there is little consistency in the terminology used to describe ITS studies ^15, 16^, our search terms are intentionally broad to achieve greater search sensitivity. Terms will be searched both as free text in the titles, abstracts and keywords fields, and as MeSH terms (or equivalent) where applicable. The MEDLINE (Ovid) strategy is presented in Table 1, and the search strategies for the remaining databases are presented in Appendix 1 (see *Extended data*) ^20^. The search is limited to the period 1 Jan 2000 to 11 Oct 2019 for all databases except CDSR which is limited to the period 1 Jan 2000 to 9 Aug 2019.

**Table 1.** MEDLINE (Ovid) search strategy.

#	Searches
1	Interrupted Time Series Analysis/
2	interrupted time series.mp.
3	(time series or time trend$ or trend analys?s).mp.
4	(change point or repeated measures or phase design or multiple baseline$ or difference-in-difference$ or single case research or single case experimental).mp.
5	(ARIMA or autoregressive integrated moving average or integrated moving average or piecewise regression or segmented regression).mp.
6	or/1–5
7	(Systematic Review or Meta-Analysis).pt. or (meta-analys?s or pooled analys?s).ti,ab,kw.
8	and/6–7
9	Limit 8 to (abstracts and English language and yr=”2000-Current”)

### Study selection

Citations identified from the searches will be imported into Endnote X8 (Clarivate Analytics, Philadelphia) to remove duplicates. Titles and abstracts will be sorted by year in descending order and will be screened against the eligibility criteria, with each abstract assessed as: 1) ‘Yes/Maybe includes two or more ITS studies’ and ‘Yes/Maybe a meta-analysis of ITS studies has been undertaken’, 2) ‘Yes/Maybe includes two or more ITS studies’ and ‘No meta-analysis of ITS studies has been undertaken’, or 3) ‘No, does not include two or more ITS studies’. This process will be piloted on 20 studies by EK, SLT, AK and JEM. The remaining abstracts will be screened independently by at least two members of the review team (EK, and any of SLT, AK and JEM). The full-text articles of the titles and abstracts assessed as potentially meeting the eligibility criteria (i.e. group 1 above) will be retrieved, sorted by most recent first and screened against the eligibility criteria until all reviews (if less than 100), or the 100 most recently published reviews are identified. Conflicts in screening decisions at the abstract and full-text stages will be resolved via discussion between the screeners or through consultation with the broader team.

### Sample size

Our sample size of 100 reviews was primarily selected for reasons of feasibility. A sample of this size will allow estimation of the percentage of reviews with a particular element (e.g. the prevalence of the reviews that re-analyse the primary study data) to within a maximum margin of error of 10% (assuming a prevalence of 50%). This margin of error represents the worst-case scenario and will decrease if the prevalence varies from 50%.

### Selection of outcomes

Reviews may include several meta-analyses of ITS studies for *different* outcomes. We plan to examine the meta-analysis methods for only *one* outcome per review. The following set of rules will be applied hierarchically until a unique outcome is identified (for which there could be multiple meta-analyses of different effect estimates):

1) The outcome that has the largest number of effect measures (e.g. the outcome that has meta-analyses of level change and slope change estimates would be selected ahead of an outcome with only a meta-analysis of level change estimates);
2) The outcome with the largest number of ITS studies; or
3) The outcome that is first reported in the abstract, then the methods section, then the results section of the manuscript.

A single outcome is chosen as it is likely that the meta-analysis methods are consistent across outcomes within a review. Criterion 1 has been included so that we can capture the range of effect measures used. Uncertainty in the selection of the outcome will be resolved through discussion with the review team.

### Data extraction and management

The data extraction form will be designed using the Research Electronic Data Capture (REDCap) online designer ^21, 22^. The review team (EK, AK, SLT, ABF, and JEM) will pilot the data extraction form by independently extracting data from 10 reviews. This pilot testing will be used to revise the form if we uncover ambiguity or a lack of clarity in any items, identify missing items and test the logic of the form. Following piloting, data extraction will be undertaken by EK for all eligible studies and independently by at least two members of the review team (one of AK, SLT, ABF and JEM) for a further 20% of randomly selected reviews. Any inconsistencies in data extraction will be resolved via discussion between the data extractors or through consultation with the broader team. For any items where a large percentage of inconsistency is found, the percentage of studies with double data extraction will be increased.

A summary of the data extraction items is presented in Table 2, while version 1 of the data extraction form can be viewed in Appendix 2 (see *Extended data*) ^23^. In brief, we will extract details of the review’s aims, meta-analysis methods (including the reviewer’s rationale) and methods used to assess the methodological quality and/or risk of bias of the included ITS studies. For the selected outcome, we will extract the type of effect measure(s), methods of synthesis, adjustment for autocorrelation and/or seasonality.

**Table 2.** Summary of data extraction items for the selected meta-analysis.

Domain	Items
**Review characteristics**	Author name; publication year; journal; target population or content area; number of ITS studies included in the review; reviewers’ definition of ITS studies
**Outcome and studies included**	Description and type of outcome (e.g. continuous, count, rate); number of ITS studies measuring the chosen outcome
**Methods for synthesising ITS results**	Number of ITS studies meta-analysed; use of primary study data (i.e. re-analysis); pairwise or network meta- analysis; fixed/random effects model; methods to quantify between-study variation
**Results/Estimates**	Description and type of effect measures (e.g. change in level, change in slope, combination of change in level and slope (i.e., counterfactual)); completeness of reporting estimates (e.g. combined effect estimate, confidence interval, measure of heterogeneity)
**Risk of bias and/or assessment of study quality**	Description of assessment (if performed) of primary study risk of bias / methodological quality; tool or domains used for assessment

Abbreviations: ITS, interrupted time series

### Analysis

We will summarise the characteristics of included systematic reviews with descriptive statistics. For categorical data (e.g. the meta-analysis approach used, the risk of bias tool used) we will present frequencies (with percentages), and for numerical data (e.g. the number of meta-analysed ITS studies, the number of pooled estimates) we will present means (with standard deviations) or medians (with interquartile range). Statistical analyses will be undertaken using Stata version 15.0 ^24^.

## Discussion

To our knowledge, this will be the first review to examine methods for meta-analysis of ITS studies that are used in practice. Specifically, the choice of meta-analysis approach, effect measures, completeness of reporting, and tools for assessing quality or risk of bias (if undertaken). The results of this review will inform our broader research program which aims to examine how different meta-analysis methods of ITS studies perform and provide guidance on the methods. Specifically, the review will identify the range of statistical methods that are used in practice, characteristics of the times series studies included in the meta-analyses (e.g. number of series, length of series) and the types of effect measures used (e.g. level change, slope change). These characteristics will inform a statistical simulation study that will examine the performance of different methods for meta-analysis of ITS studies. In addition, we will identify reviews for which the raw time series data of the included ITS studies are reported. These reviews will be used in an empirical evaluation to examine the impact of using different meta-analysis methods of real ITS studies.

### Strengths and limitations

There are several strengths to this study. The search, screening and data extraction methods have been prespecified, and the study has been registered with PROSPERO (accepted 28 Apr 2020 submitted 4 Oct 2019). Further, we will search a broad range of databases, encompassing the areas of health, economics, psychology and education. This will allow us to identify a broader range of meta-analysis methods in use, not restricted to a particular discipline.

While the study will be limited by our ability to identify all potentially eligible reviews and meta-analyses of ITS studies, our search strategy attempts to capture the various ways these studies are described. However, given ITS studies are often not identified as such ^16^ and that our search is restricted to articles written in English, it is likely that we will not capture all reviews and meta-analyses that include ITS studies. Conversely, we may end up including reviews where no information regarding the definition of the included ITS studies is provided, or where an inappropriate label of ITS has been applied to included studies. While we will not exclude these reviews, we will record the reviewers’ definition of an ITS study.

## Conclusions

The ITS design is often used to examine the effects of organisational, policy change or public health interventions or exposures. Meta-analysis of results from these studies provides the opportunity to estimate the interruption’s impact more precisely, and investigate factors that may modify the size of the impact. However, there is a paucity of guidance available for meta-analysing results from ITS studies. Results from this review will provide the first examination of meta-analysis methods used in practice to combine results from ITS studies. This will be used to inform future research that investigates how different methods perform, from which guidance will be developed.

## Data availability

### Underlying data

No underlying data are associated with this article.

### Extended data

Figshare (Monash University repository, known as Bridges): Methods used to meta-analyse results from interrupted time series studies: A methodological systematic review protocol - Appendix 1 Search strategy. https://doi.org/10.26180/5e3b5cc4acf30 ^20^.

Figshare (Monash University repository, known as Bridges): Methods used to meta-analyse results from interrupted time series studies: A methodological systematic review protocol – Appendix 2 Data extraction form (version 1). https://doi.org/10.26180/5f0682e008dda ^23^.

### Reporting guidelines

Figshare (Monash University repository, known as Bridges): PRISMA-P checklist for ‘Methods used to meta-analyse results from interrupted time series studies: A methodological systematic review protocol’. https://doi.org/10.26180/5e3b5d75c00a6 ^25^.

Data are available under the terms of the Creative Commons Attribution 4.0 International license (CC-BY 4.0).

## References

[1] Higgins JP Ramsay C Reeves BC: Issues relating to study design and risk of bias when including non-randomized studies in systematic reviews on the effects of interventions. *Res Synth Methods.* 2013;4(1):12–25. 10.1002/jrsm.1056 26053536

[2] Wagner AK Soumerai SB Zhang F: Segmented regression analysis of interrupted time series studies in medication use research. *J Clin Pharm Ther.* 2002;27(4):299–309. 10.1046/j.1365-2710.2002.00430.x 12174032

[3] Watson SI Dixon-Woods M Taylor CA: Revising ethical guidance for the evaluation of programmes and interventions not initiated by researchers. *J Med Ethics.* 2020;46(1):26–30. 10.1136/medethics-2018-105263 31481472 PMC6984058

[4] Shadish WR Cook TD Campbell DT: Experimental and quasi-experimental designs for generalized causal inference.2002 Reference Source

[5] Bernal JL Cummins S Gasparrini A: The use of controls in interrupted time series studies of public health interventions. *Int J Epidemiol.* 2018;47(6):2082–93. 10.1093/ije/dyy135 29982445

[6] McKenzie JE Beller EM Forbes AB: Introduction to systematic reviews and meta-analysis. *Respirology.* 2016;21(4):626–37. 10.1111/resp.12783 27099100

[7] Deeks JJ: Systematic reviews of published evidence: miracles or minefields? *Ann Oncol.* 1998;9(7):703–9. 10.1023/a:1008335706631 9739434

[8] Deeks J Higgins J Altman D: Chapter 10: Analysing data and undertaking meta-analyses.In: *Cochrane Handbook for Systematic Reviews of Interventions* Cochrane.2019 10.1002/9781119536604.ch10

[9] Caldwell DM Dias S Welton NJ: Extending Treatment Networks in Health Technology Assessment: How Far Should We Go? *Value Health.* 2015;18(5):673–81. 10.1016/j.jval.2015.03.1792 26297096 PMC4553939

[10] Thompson SG Higgins JP: How should meta-regression analyses be undertaken and interpreted? *Stat Med.* 2002;21(11):1559–73. 10.1002/sim.1187 12111920

[11] Gebski V Ellingson K Edwards J: Modelling interrupted time series to evaluate prevention and control of infection in healthcare. *Epidemiol Infect.* 2012;140(12):2131–41. 10.1017/S0950268812000179 22335933 PMC9152341

[12] DerSimonian R Laird N: Meta-analysis in clinical trials. *Control Clin Trials.* 1986;7(3):177–88. 10.1016/0197-2456(86)90046-2 3802833

[13] Ramsay CR Matowe L Grilli R: Interrupted time series designs in health technology assessment: lessons from two systematic reviews of behavior change strategies. *Int J Technol Assess Health Care.* 2003;19(4):613–23. 10.1017/s0266462303000576 15095767

[14] Jandoc R Burden AM Mamdani M: Interrupted time series analysis in drug utilization research is increasing: systematic review and recommendations. *J Clin Epidemiol.* 2015;68(8):950–6. 10.1016/j.jclinepi.2014.12.018 25890805

[15] Hudson J Fielding S Ramsay CR: Methodology and reporting characteristics of studies using interrupted time series design in healthcare. *BMC Med Res Methodol.* 2019;19(1):137. 10.1186/s12874-019-0777-x 31272382 PMC6609377

[16] Polus S Pieper D Burns J: Heterogeneity in application, design, and analysis characteristics was found for controlled before-after and interrupted time series studies included in Cochrane reviews. *J Clin Epidemiol.* 2017;91:56–69. 10.1016/j.jclinepi.2017.07.008 28750849

[17] Huitema BE McKean JW: Identifying autocorrelation generated by various error processes in interrupted time-series regression designs - A comparison of AR1 and portmanteau tests. *Educ Psychol Meas.* 2007;67(3):447–59. 10.1177/0013164406294774

[18] Turner SL Karahalios A Forbes AB: Design characteristics and statistical methods used in interrupted time series studies evaluating public health interventions: A review. *J Clin Epidemiol.* 2020;122:1–11. 10.1016/j.jclinepi.2020.02.006 32109503

[19] Turner SL Karahalios A Forbes AB: Design characteristics and statistical methods used in interrupted time series studies evaluating public health interventions: protocol for a review. *BMJ Open.* 2019;9(1):e024096. 10.1136/bmjopen-2018-024096 30696676 PMC6352832

[20] Korevaar E Karahalios E Forbes A: Methods used to meta-analyse results from interrupted time series studies: A methodological systematic review protocol - Appendix 1 Search strategy. *figshare.*Journal contribution.2020 10.26180/5e3b5cc4acf30 PMC7607479 33163155

[21] Harris PA Taylor R Minor BL: The REDCap consortium: Building an international community of software platform partners. *J Biomed Inform.* 2019;95:103208. 10.1016/j.jbi.2019.103208 31078660 PMC7254481

[22] Harris PA Taylor R Thielke R: Research electronic data capture (REDCap)--a metadata-driven methodology and workflow process for providing translational research informatics support. *J Biomed Inform.* 2009;42(2):377–81. 10.1016/j.jbi.2008.08.010 18929686 PMC2700030

[23] Korevaar E Karahalios E Forbes A: Methods used to meta-analyse results from interrupted time series studies: A methodological systematic review protocol – Appendix 2 Data extraction form (version 1). *figshare.*Journal contribution.2020. 10.26180/5f0682e008dda PMC7607479 33163155

[24] StataCorp: Stata statistical software: release 15.Tx: College Station StataCorp LLC;2017.

[25] Korevaar E Karahalios E Forbes A: Methods used to meta-analyse results from interrupted time series studies: A methodological systematic review protocol - Completed PRISMA-P checklist. *figshare.*Journal contribution.2020 10.26180/5e3b5d75c00a6 PMC7607479 33163155

