# Supplementary material for: Methods used to meta-analyse results from interrupted time series studies: A methodological systematic review protocol
Source: F1000Res. 2020 Dec 11;9:110. Originally published 2020 Feb 12. [Version 3] doi: 10.12688/f1000research.22226.3 (PMC7607479; doi:10.12688/f1000research.22226.3)
Supplement: Peer review history

# Peer review: Reviewer response for version 2

**DOI:** 10.5256/f1000research.27594.r73704

**Published:** 2020-11-02

**Authors:** Tim Mathes

**Affiliations:** 1 Institute for Research in Operative Medicine, Faculty of Health, School of Medicine, Witten/Herdecke University, Cologne, Germany

This is a very interesting and well-written study protocol. I have only some minor suggestions for consideration.

- The search strategy assumes that the very specific study type/design label ITS is reported in the abstract of the meta-analysis. I recognized that this problem is already addressed as limitation. However, I suppose that an additional related problem would be that the search will result in an imbalance towards EPOC-Cochrane because in my experience this usually mention ITS in the abstract in contrast to other non-EPOC SR. It might be worth to adapted the sampling strategy accordingly, e.g. screening until 50 Cochrane and 50 non-Cochrane reviews.
- The search includes the term “ARIMA” but no other terms that specify possible analysis of ITS (e.g. segmented regression) and consequently would probably be biased towards ITS that are analysed using ARIMA approaches.
- “We will not restrict the meta-analysis by approach, that is, we will include both one-stage and two-stage meta-analyses. We will only include meta-analyses that combine estimates of model parameters, or combinations of these (e.g. pre-intervention fitted trend, slope change, level change)”. This statement is contradictory. Pooled estimates of one-stage models are based on pseudo IPD that are pooled in one-step. Thus, there is no model parameter for each study and consequently it will not give model parameters, that can be combined (second step in two-stage models).
- “the study is a review that includes at least two ITS studies which meet the review authors’ definition of an ITS design”. Does it mean that you will also include SR which include ITS with a control group? If yes, I think the meta-analysis methods must be analysed separately.

Is the study design appropriate for the research question?

Yes

Is the rationale for, and objectives of, the study clearly described?

Yes

Are sufficient details of the methods provided to allow replication by others?

Yes

Are the datasets clearly presented in a useable and accessible format?

Yes

Reviewer Expertise:

medical statistics, research synthesis methods, non-randomized studies

I confirm that I have read this submission and believe that I have an appropriate level of expertise to confirm that it is of an acceptable scientific standard.

# Response

**Published:** 2020-12-08

**Authors:** Elizabeth Korevaar

**Affiliations:** Monash University, Australia

**We would like to thank Dr Mathes for their feedback on our protocol and the suggestions for its improvement. Below, we have addressed each of the four items raised.**

**Minor comments:**

 This is a very interesting and well-written study protocol. I have only some minor suggestions for consideration.

 1. The search strategy assumes that the very specific study type/design label ITS is reported in the abstract of the meta-analysis. I recognized that this problem is already addressed as limitation. However, I suppose that an additional related problem would be that the search will result in an imbalance towards EPOC-Cochrane because in my experience this usually mention ITS in the abstract in contrast to other non-EPOC SR. It might be worth to adapted the sampling strategy accordingly, e.g. screening until 50 Cochrane and 50 non-Cochrane reviews.

**Thank you for your interest in our protocol and your suggestions. As noted, we acknowledged that study labels used by the systematic reviewers are a limitation of our search strategy. We attempted to circumvent this limitation by including broader, related terms (e.g. repeated measures, difference-in-difference) in our search strategy. We appreciate the suggestion of adapting the sampling strategy to increase the proportion of non-EPOC reviews. However, as the search and screening has been completed and has identified a total of 54 eligible reviews, we have extracted data from all eligible reviews.**

 2. The search includes the term “ARIMA” but no other terms that specify possible analysis of ITS (e.g. segmented regression) and consequently would probably be biased towards ITS that are analysed using ARIMA approaches.

**In line 5 of our search strategy (Table 1), as noted by the reviewer, we have included the terms *ARIMA*, *autoregressive integrated moving average*, and *integrated moving average*. Additionally, this line in our search strategy includes the terms *piecewise regression* and *segmented regression*.**

 3. “We will not restrict the meta-analysis by approach, that is, we will include both one-stage and two-stage meta-analyses. We will only include meta-analyses that combine estimates of model parameters, or combinations of these (e.g. pre-intervention fitted trend, slope change, level change)”. This statement is contradictory. Pooled estimates of one-stage models are based on pseudo IPD that are pooled in one-step. Thus, there is no model parameter for each study and consequently it will not give model parameters, that can be combined (second step in two-stage models).

**To address the ambiguity in our target effect measures, we have amended the wording of the highlighted text to “ *We will not restrict the meta-analysis by approach, that is, we will include both one-stage and two-stage meta-analyses. We will only include meta-analyses that provide pooled estimates of model parameters that quantify the effect of the interruption*”. The pooled estimates of model parameters that we are interested in are those that quantify the effect of the interruption (whether that be parameterised as a level change, slope change, etc). We are not interested in the effects of other covariates either in a one-stage model or in the first stage of a two-stage approach.**

 4. “the study is a review that includes at least two ITS studies which meet the review authors’ definition of an ITS design”. Does it mean that you will also include SR which include ITS with a control group? If yes, I think the meta-analysis methods must be analysed separately.

**Yes, we will include systematic reviews that include a meta-analysis of two or more ITS studies. The studies could all have an interruption (i.e. uncontrolled ITS) or at least one of the series might be a control series (i.e. controlled ITS). Controlled time series can be included by using a unified model (i.e., a one-stage meta-analysis, or the first step of a two-stage meta-analysis), a statistical comparison (i.e., separate analyses of the interrupted series and control series, with a statistical comparison of the two) or a narrative comparison. Our data collection tool will capture information on which of the three above scenarios applies to the meta-analysis, how many are controlled versus uncontrolled, and how the controlled studies were incorporated into the review.**

**Kind regards,**

**Elizabeth Korevaar**

# Peer review: Reviewer response for version 1

**DOI:** 10.5256/f1000research.24513.r62669

**Published:** 2020-05-20

**Authors:** Alexandra McAleenan

**Affiliations:** 1 Population Health Sciences, Bristol Medical School, University of Bristol, Bristol, UK

This article is a protocol for a systematic review that aims to describe the meta-analysis methods (and some of the systematic review methods) used in systematic reviews that include interrupted time series (ITS) studies.

 The protocol is thorough and well written. A comprehensive search of the literature will be undertaken to identify reviews of health, public health, psychology, education and economic interventions. There are clear eligibility criteria for reviews, and the data to be extracted is well described, as is the planned analysis.

 I have a couple of minor suggestions. In the abstract it would be preferable to state how the 100 reviews will be selected (for e.g. "We will identify up to the 100 most recently published reviews"). You could also add in that you will be looking at the tools used to assess bias/methodological quality of ITS studies (which one could argue is more looking at systematic review methods that meta-analysis methods).

 You could also clarify, by adding to the summary of data extraction items, that you will be looking at the review authors' definition of ITS studies. I presume you will also be extracting whether any of the ITS studies included a control group, and perhaps how the control group was used (especially if any re-analysis was undertaken)?

Is the study design appropriate for the research question?

Yes

Is the rationale for, and objectives of, the study clearly described?

Yes

Are sufficient details of the methods provided to allow replication by others?

Yes

Are the datasets clearly presented in a useable and accessible format?

Not applicable

Reviewer Expertise:

Systematic reviewing.

I confirm that I have read this submission and believe that I have an appropriate level of expertise to confirm that it is of an acceptable scientific standard.

# Response

**Published:** 2020-07-09

**Authors:** Elizabeth Korevaar

**Affiliations:** Monash University, Australia

**We would like to thank Dr McAleenan for their feedback on our protocol and the suggestions for its improvement. Below, we have addressed each of the suggestions raised.**

**Minor suggestions:**

1. In the abstract it would be preferable to state how the 100 reviews will be selected (for e.g. "We will identify up to the 100 most recently published reviews"). You could also add in that you will be looking at the tools used to assess bias/methodological quality of ITS studies (which one could argue is more looking at systematic review methods that meta-analysis methods).

** We have revised the ‘Abstract’ to make the selection process clearer:**

**“We will identify *the* 100 *most recent* reviews *(published between 1 January 2000 and 11 October 2019)* that include meta-analyses of ITS studies from a search of eight electronic databases covering several disciplines (public health, psychology, education, economics).”**

**In addition, we have added text to note we will be extracting information about the tools used to assess risk of bias/methodological quality:**

**“From eligible reviews we will extract details at the review level including discipline, type of interruption *and any tools used to assess the risk of bias / methodological quality of included ITS studies*; at the meta-analytic level we will extract type of outcome, effect measure(s), meta-analytic methods, and any methods used to re-analyse the individual ITS studies. Descriptive statistics will be used to summarise the data.”**

  

1. You could also clarify, by adding to the summary of data extraction items, that you will be looking at the review authors' definition of ITS studies. I presume you will also be extracting whether any of the ITS studies included a control group, and perhaps how the control group was used (especially if any re-analysis was undertaken)?

**We have added “reviewers’ definition of ITS studies” to the summary of data extraction items table (Table 2). In addition, we have included the current version of our data extraction form as an additional file in the ‘Extended data’ section. We will collect data on whether and how control series were incorporated into the meta-analysis (see rows 69-71 of the data extraction form, Appendix 2).**

**Kind regards,**

**Elizabeth Korevaar**

# Peer review: Reviewer response for version 1

**DOI:** 10.5256/f1000research.24513.r60103

**Published:** 2020-02-25

**Authors:** Christopher James Rose

**Affiliations:** 1 Norwegian Institute of Public Health, Oslo, Norway

In this protocol, the authors propose to systematically survey methods used to meta-analyze results from interrupted time series (ITS) studies. ITS studies are particularly useful in systematic reviews (SRs) of interventions that cannot be studied using randomized trials (e.g., due to practical, ethical, or legal reasons). Briefly, the protocol plans to identify 100 recent systematic reviews that meta-analyze results from ITS studies and then extract and summarize characteristics of the methods used.

 I agree with the authors that we currently know relatively little about the characteristics of methods used in meta-analyses of ITS results. From my point of view, I would like to have evidence that can be used to help design SRs that will meta-analyze ITS results, and to help understand potential weaknesses of such SRs. For example, I would like to have evidence to inform the number of pre- and post-interruption time points that should be required of ITS studies for inclusion in a meta-analysis, and information about whether certain ITS designs and analysis methods lead to excessive bias or imprecision. The protocol says that the resulting work will inform subsequent research that addresses these kinds of questions, using simulation and empirical evaluation. My understanding is that knowing about the landscape of methods that have been used in recent SRs will allow this subsequent work to address relevant questions (e.g., it could allow the authors to design simulation studies that usefully model what is being done in practice).

 My review focuses mainly on the conceptual and statistical aspects of the protocol. I cannot comment on other aspects such as the literature search.

 I have two "major" criticisms of the protocol:

1. Because I am more interested in the evidence that the subsequent research will hopefully deliver, I would like to see more detailed thought in this protocol about how the subsequent work will be performed. This should then inform what the product of the present protocol needs to deliver to ensure the success of the subsequent work. Perhaps this has already been thought through in detail and not presented here. However, if this work has not been done, I encourage the authors to do it and update the protocol.
2. The analysis is not planned in enough detail that it could be implemented without having to make important choices after having seen the data. I think this potentially leads to at two problems. First, being able to choose from among several possible analyses and means of presentation risks introducing bias. Second, more detailed planning at the protocol stage may prevent problems that would otherwise only become apparent while the work is being done. I suggest that the authors substantially revise this section to specify in detail what they will do and how they will report their results, including preparing skeleton tables and/or figures. I think that thinking through these issues at the protocol stage will likely make this and subsequent work (see point 1 above) more efficient, and lead to higher quality papers.

I have the following "minor" comments and suggestions:

1. I suggest that the abstract is clarified to say that the authors will study the 100 **most recent** systematic reviews that include ITS analyses (and include the date range), rather than simply saying 100 (or whatever the final sample size is determined to be). My concern when reading the abstract was that the 100 SRs could be chosen arbitrarily, giving rise to potential bias.
2. It would be useful for the authors to clarify that, with respect to estimating a binomial proportion, their proposed sample size of 100 would give them a worst-case margin of error of **plus or minus** approximately ten percentage points (i.e., 40.2% to 59.8%), if the population parameter is 50%.
3. This margin of error is actually quite wide. I wonder if the authors have considered how plausible it is that the population parameter will often be close to the worst-case of 50%, and if so, whether the relatively wide confidence intervals will be informative enough for their subsequent work that will build on this paper?
4. The sample size of 100 seems to have been chosen under the assumption that a binomial proportion will be estimated for each factor studied (i.e., that each factor will have two levels). However, many of the criteria specified are factors with more than two levels (e.g., the protocol gives the example of three types of outcome that included reviews may study: continuous, count, and rate). Given that, I would encourage the authors to think about the more general case of estimating multinomial proportions. This would require a larger sample size for a worst-case scenario equivalent to that of a binomial distribution. A quick search identified Thompson 1987 ^1^, which provides a table for estimating sample size for estimating multinomial proportions. Briefly, if the authors want to estimate multinomial proportions with 95% CIs that give a margin or error of plus or minus 10%, that paper shows that the authors should include at least 128 studies (irrespective of the number of levels of the factor studied). However, I encourage the authors to double-check this informal power analysis. The authors would then also need to use an appropriate method to estimate the proportions and their confidence intervals. Given the authors plan to use Stata, I think the correct way to do this is to estimate the proportions using syntax like " **mlogit myvar**" and then obtain the estimates and their confidence intervals via " **margins, predict()"**, but again I suggest the authors double-check this.
5. With respect to the "Selection of outcomes" section, it may be useful to rewrite item 1 in terms of "number of model parameters" rather than "number of effect measures". The meaning of the current text was not immediately clear to me.
6. I suggest the authors plan to extract a little more detail about the methods used to analyze ITS studies, so that this can be used to inform subsequent work. For example, I am interested to know how often "incorrect" model assumptions are assumed, and under what conditions meaningfully incorrect conclusions result from meta-analyses that include ITS results from misspecified models. I'm thinking about the case where an outcome is bounded, where the observed pre- and/or post-interruption levels are close to the bounds, and the analysis assumes an error distribution whose domain includes values outside the bounds. For example, imagine the outcome "percentage of prescriptions with dosage errors", which is bounded to [0%, 100%]. If a normal error distribution is assumed in the ITS analysis, that distribution would incorrectly permit outcomes <0% and >100%. This may not be a problem if the residual standard deviation is small relative to the distance from the mean to the nearest bound, but if the observed data were close to 0% and/or 100% and the residual standard deviation was sufficiently large, the estimate of relative treatment effect estimate might be biased. I would then be interested in knowing, via empirical or simulation work from the planned subsequent research, whether these kinds of errors lead to meaningfully incorrect conclusions when such ITS results are brought into meta-analyses. If this kind of misspecification is uncommon, then perhaps that subsequent work is unnecessary, but my suspicion is that model misspecification (and this misspecification in particular) is actually very common.
7. I'm also interested in knowing what proportion of ITSs included in meta-analyses assume ITS models that are misspecified in terms of being overly simplistic (e.g., piecewise constant time series with an instantaneous post-intervention step-change). The protocol plans to survey which ITS models are used, but it's not clear whether it aims to judge whether the use is appropriate or not. This is to some extent a judgement about whether particular models are appropriate in particular contexts (all models are approximations of reality), but it would be useful to have a solid evidence base on which to make recommendations for what people should consider when they undertake ITS-based SRs.
8. The protocol talks about ITS analyses that include versus exclude controls (i.e., where the interruption does not occur), but it's not clear that the protocol will extract data on this. It would be interesting to know what proportion of SRs of ITS studies permit or include uncontrolled ITS results, and ultimately to have some information about whether inclusion of such studies leads to meaningfully incorrect conclusions (I assume it does, though I could imagine that it may be possible to include controlled and uncontrolled ITS results in a meta-regression and still reasonably estimate treatment effect). Similarly, it would be interesting to know how many controls are used (i.e., I assume that ITSs with one control are common but that 10 or 20 controls are quite rare), and then from the subsequent research, the number of controls that SR protocols should specify.
9. I suggest including the restriction to English as a possible limitation.

I wish the authors success with their research!

Is the study design appropriate for the research question?

Yes

Is the rationale for, and objectives of, the study clearly described?

Yes

Are sufficient details of the methods provided to allow replication by others?

Partly

Are the datasets clearly presented in a useable and accessible format?

Not applicable

Reviewer Expertise:

Statistical modelling, biomedical research

I confirm that I have read this submission and believe that I have an appropriate level of expertise to confirm that it is of an acceptable scientific standard, however I have significant reservations, as outlined above.

## References

[1] : Sample Size for Estimating Multinomial Proportions. *The American Statistician*.1987;41(1) : 10.1080/00031305.1987.10475440 42-46 10.1080/00031305.1987.10475440

# Response

**Published:** 2020-07-09

**Authors:** Elizabeth Korevaar

**Affiliations:** Monash University, Australia

**We would like to thank Dr Rose for their feedback on our protocol and the suggestions for its improvement. Below, we have addressed each of the items raised.**

**Major comments:**

My review focuses mainly on the conceptual and statistical aspects of the protocol. I cannot comment on other aspects such as the literature search.

1. Because I am more interested in the evidence that the subsequent research will hopefully deliver, I would like to see more detailed thought in this protocol about how the subsequent work will be performed. This should then inform what the product of the present protocol needs to deliver to ensure the success of the subsequent work. Perhaps this has already been thought through in detail and not presented here. However, if this work has not been done, I encourage the authors to do it and update the protocol.

**Thank you for your interest in our study and our future planned research. In this manuscript, we present the protocol for a systematic review of meta-analysis methods used to combine results from interrupted time series studies. While the results of our systematic review will inform subsequent research, outlining details of the future projects is beyond the scope of the current manuscript. However, we have provided more detail about the planned statistical simulation and empirical evaluation in the first paragraph of the ‘Discussion’: “The results of this review will inform our broader research program which aims to examine how different meta-analysis methods of ITS studies perform *and provide guidance on the methods. Specifically, the review will identify the range of statistical methods that are used in practice, characteristics of the times series studies included in the meta-analyses (e.g. number of series, length of series) and the types of effect measures used (e.g. level change, slope change). These characteristics will inform a statistical simulation study that will examine the performance of different methods for meta-analysis of ITS studies. In addition, we will identify reviews for which the raw time series data of the included ITS studies are reported. These reviews will be used in an empirical evaluation to examine the impact of using different meta-analysis methods of real ITS studies.*”. In addition, we have provided the current version of our data extraction form as an additional file (Appendix 2) in the ‘Extended data’ section. The data extraction form provides details of the data to be extracted and summarised.**

2. The analysis is not planned in enough detail that it could be implemented without having to make important choices after having seen the data. I think this potentially leads to at two problems. First, being able to choose from among several possible analyses and means of presentation risks introducing bias. Second, more detailed planning at the protocol stage may prevent problems that would otherwise only become apparent while the work is being done. I suggest that the authors substantially revise this section to specify in detail what they will do and how they will report their results, including preparing skeleton tables and/or figures. I think that thinking through these issues at the protocol stage will likely make this and subsequent work (see point 1 above) more efficient, and lead to higher quality papers.

**We have added further detail in the ‘Analysis’ section about the general table structure we plan to use to present the results (see following). In addition, we provide the data extraction form which includes all the items we are collecting.**

**“We will summarise the characteristics of included systematic reviews with descriptive statistics. For categorical data (e.g. the meta-analysis approach used, the risk of bias tool used) we will present frequencies (with percentages), and for numerical data (e.g. the number of meta-analysed ITS studies, the number of pooled estimates) we will present means (with standard deviations) or medians (with interquartile range).”**

**The items (their response options – see the data extraction form in Appendix 2) and summary statistics will be grouped into tables using a structure such as the following: **

1. **General study characteristics (e.g. research disciplines; types of interventions; definition used to classify interrupted time series; risk of bias or methodological quality assessment of included studies undertaken)**
2. **Included study characteristics (e.g. number of included ITS studies; average number of points pre- and post-interruption)**
3. **Meta-analysis methods (e.g. justification given for chosen meta-analysis method; re-analysis of primary study data undertaken, one-stage or two-stage meta-analysis used, chosen meta-analysis model, heterogeneity estimator used)**
4. **Effect measures used (e.g. number of effect measure, which effect measures, standardisation of the effect measure)**
5. **Additional analyses (e.g. sensitivity analyses, subgroup analyses)**
6. **Completeness of reporting (e.g. reporting of combined effect, reporting of measure of precision)**

**Minor comments:**

1. I suggest that the abstract is clarified to say that the authors will study the 100 most recent systematic reviews that include ITS analyses (and include the date range), rather than simply saying 100 (or whatever the final sample size is determined to be). My concern when reading the abstract was that the 100 SRs could be chosen arbitrarily, giving rise to potential bias.

**We have revised the text in the ‘Abstract’ as follows:**

**“We will identify the 100 most recent reviews (published between 1 January 2000 and 11 October 2019) that include meta-analyses of ITS studies from a search of eight electronic databases covering several disciplines (public health, psychology, education, economics).”**

2. It would be useful for the authors to clarify that, with respect to estimating a binomial proportion, their proposed sample size of 100 would give them a worst-case margin of error of **plus or minus** approximately ten percentage points (i.e., 40.2% to 59.8%), if the population parameter is 50%.

**We have revised the text in the ‘Sample size’ section to:**

**“Our sample size of 100 reviews was primarily selected for reasons of feasibility. A sample of this size will allow estimation of the percentage of reviews with a particular element (e.g. the prevalence of the reviews that re-analyse the primary study data) to within a maximum margin of error of 10% (assuming a prevalence of 50%). This margin of error represents the worst-case scenario and will decrease if the prevalence varies from 50%.”**

3. This margin of error is actually quite wide. I wonder if the authors have considered how plausible it is that the population parameter will often be close to the worst-case of 50%, and if so, whether the relatively wide confidence intervals will be informative enough for their subsequent work that will build on this paper?

**We believe that our sample size will generally be sufficient, such that our interpretation of the confidence interval limits will be consistent. For example, if we found that the percentage of reviews that use a particular method was 10% (95%CI: 4% to 16%), our interpretation of the limits of the confidence interval would lead to the same conclusion that the method was not commonly used. As another example, if the outcome was complete reporting of effect estimates (i.e., reporting the effect estimate and a measure of precision), and 50% of the reviews were found to completely report the effect, our interpretation would not differ at the limits of the confidence interval; if the true percentage was 40% or 60%, we would be concerned.**

4. The sample size of 100 seems to have been chosen under the assumption that a binomial proportion will be estimated for each factor studied (i.e., that each factor will have two levels). However, many of the criteria specified are factors with more than two levels (e.g., the protocol gives the example of three types of outcome that included reviews may study: continuous, count, and rate). Given that, I would encourage the authors to think about the more general case of estimating multinomial proportions. This would require a larger sample size for a worst-case scenario equivalent to that of a binomial distribution. A quick search identified Thompson 1987 ^1^, which provides a table for estimating sample size for estimating multinomial proportions. Briefly, if the authors want to estimate multinomial proportions with 95% CIs that give a margin or error of plus or minus 10%, that paper shows that the authors should include at least 128 studies (irrespective of the number of levels of the factor studied). However, I encourage the authors to double-check this informal power analysis. The authors would then also need to use an appropriate method to estimate the proportions and their confidence intervals. Given the authors plan to use Stata, I think the correct way to do this is to estimate the proportions using syntax like **"mlogit myvar"** and then obtain the estimates and their confidence intervals via **"margins, predict()"**, but again I suggest the authors double-check this.

**As noted in the ‘Sample size’ section, our sample size of 100 reviews was primarily selected for reasons of feasibility, so we do not have the ability to increase the sample size. Importantly, however, most items are binary and not multinomial. We acknowledge that for those few multinomial items, we will have less precision.**

5. With respect to the "Selection of outcomes" section, it may be useful to rewrite item 1 in terms of "number of model parameters" rather than "number of effect measures". The meaning of the current text was not immediately clear to me.

**We have chosen the term “effect measures” since this is the terminology used in the Cochrane Handbook (Chapter 10: Analysing data and undertaking meta-analyses). The “number of model parameters” is not interchangeable with the “number of effect measures” because they have different meanings. Effect measures in ITS studies are generally computed as a combination of the model parameter estimates (e.g. area under the curve, long-term change in level).**

**Deeks JJ, Higgins JPT, Altman DG (editors). Chapter 10: Analysing data and undertaking meta-analyses. In: Higgins JPT, Thomas J, Chandler J, Cumpston M, Li T, Page MJ, Welch VA (editors). Cochrane Handbook for Systematic Reviews of Interventions version 6.0 (updated July 2019). Cochrane, 2019. Available from** **www.training.cochrane.org/handbook** **.**

6. I suggest the authors plan to extract a little more detail about the methods used to analyze ITS studies, so that this can be used to inform      subsequent work. For example, I am interested to know how often "incorrect" model assumptions are assumed, and under what conditions meaningfully incorrect conclusions result from meta-analyses that include ITS results from misspecified models. I'm thinking about the case where an outcome is bounded, where the observed pre- and/or post-interruption levels are close to the bounds, and the analysis assumes an error distribution whose domain includes values outside the bounds. For example, imagine the outcome "percentage of prescriptions with dosage errors", which is bounded to [0%, 100%]. If a normal error distribution is assumed in the ITS analysis, that distribution would incorrectly permit outcomes <0% and >100%. This may not be a problem if the residual standard deviation is small relative to the distance from the mean to the nearest bound, but if the observed data were close to 0% and/or 100% and the residual standard deviation was sufficiently large, the estimate of relative treatment effect estimate might be biased. I would then be interested in knowing, via empirical or simulation work from the planned subsequent research, whether these kinds of errors lead to meaningfully incorrect conclusions when such ITS results are brought into meta-analyses. If this kind of misspecification is uncommon, then perhaps that subsequent work is unnecessary, but my suspicion is that model misspecification (and this misspecification in particular) is actually very common.

**The issue outlined is interesting and it would be useful to examine under what conditions meaningfully incorrect conclusions result from meta-analyses that include ITS results from misspecified models in a simulation study. In our study we will document whether the reviewers re-analysed the primary study data (see row 90 of the data extraction form, Appendix 2); and which analysis methods were used for the primary studies, including whether the reviewers made a judgement about the appropriateness of the analysis methods used in the primary studies (see rows 93-104 of the data extraction form).**

7. I'm also interested in knowing what proportion of ITSs included in meta-analyses assume ITS models that are misspecified in terms of being overly simplistic (e.g., piecewise constant time series with an instantaneous post-intervention step-change). The protocol plans to survey which ITS models are used, but it's not clear whether it aims to judge whether the use is appropriate or not. This is to some extent a judgement about whether particular models are appropriate in particular contexts (all models are approximations of reality), but it would be useful to have a solid evidence base on which to make recommendations for what people should consider when they undertake ITS-based SRs.

**We will extract information on the model structure when the reviewers re-analyse the included ITS studies (see row 107 of the data extraction form, Appendix 2).  From this information, we will summarise the different model structures (e.g. level change only, slope change only). We do not plan to judge the appropriateness of the model structures given, as the reviewer pointed out, this would require content knowledge of the studies. In addition, where reviewers directly use results from the primary studies, we will collect the review authors’ judgements of the appropriateness of the methods, which could include model structure misspecification (see rows 93-98).**

8. The protocol talks about ITS analyses that include versus exclude controls (i.e., where the interruption does not occur), but it's not clear that the protocol will extract data on this. It would be interesting to know what proportion of SRs of ITS studies permit or include uncontrolled ITS results, and ultimately to have some information about whether inclusion of such studies leads to meaningfully incorrect conclusions (I assume it does, though I could imagine that it may be possible to include controlled and uncontrolled ITS results in a meta-regression and still reasonably estimate treatment effect). Similarly, it would be interesting to know how many controls are used (i.e., I assume that ITSs with one control are common but that 10 or 20 controls are quite rare), and then from the subsequent research, the number of controls that SR protocols should specify.

**We will collect details on whether and how control series were incorporated into the meta-analysis (see rows 69-71 of the data extraction form, Appendix 2). Additionally, we will capture information regarding sensitivity analysis (e.g. comparing inclusion versus exclusion of control series, if performed). However, in this study, we will not assess whether or not the conclusion of the meta-analysis would change based on the inclusion of the control series.**

9. I suggest including the restriction to English as a possible limitation.

**We have amended the following paragraph in the ‘Discussion’ section to include the English language limitation:**

**“While the study will be limited by our ability to identify all potentially eligible reviews and meta-analyses of ITS studies, our search strategy attempts to capture the various ways these studies are described. However, given ITS studies are often not identified as such ^16^, and that our search is restricted to articles written in English, it is likely that we will not capture all reviews and meta-analyses that include ITS studies. Conversely, we may end up including reviews where no information regarding the definition of the included ITS studies is provided, or where an inappropriate label of ITS has been applied to included studies. While we will not exclude these reviews, we will record the reviewers’ definition of an ITS study.”**

**Kind regards,**

**Elizabeth Korevaar**
